# Associations of Peripubertal Serum Dioxin and Polychlorinated Biphenyl Concentrations with Pubertal Timing among Russian Boys

**DOI:** 10.1289/EHP154

**Published:** 2016-05-17

**Authors:** Jane S. Burns, Mary M. Lee, Paige L. Williams, Susan A. Korrick, Oleg Sergeyev, Thuy Lam, Boris Revich, Russ Hauser

**Affiliations:** 1 Environmental and Occupational Medicine and Epidemiology Program, Department of Environmental Health, Harvard T.H. Chan School of Public Health, Boston, Massachusetts, USA; 2 Pediatric Endocrine Division, Department of Pediatrics, and; 3 Department of Cell and Developmental Biology, University of Massachusetts Medical School, Worcester, Massachusetts, USA; 4 Department of Biostatistics, and; 5 Department of Epidemiology, Harvard T.H. Chan School of Public Health, Boston, Massachusetts, USA; 6 Channing Division of Network Medicine, Department of Medicine, Brigham and Women’s Hospital, Harvard Medical School, Boston, Massachusetts, USA; 7 Department of Genomics and Human Genetics, Vavilov Institute of General Genetics, Russian Academy of Sciences, Moscow, Russia; 8 Chapaevsk Medical Association, Chapaevsk, Samara Region, Russia; 9 Gradient, Cambridge, Massachusetts, USA; 10 Institute for Forecasting, Russian Academy of Sciences, Moscow, Russia

## Abstract

**Background:**

Dioxins, furans, and polychlorinated biphenyls (PCBs), dioxin-like and non-dioxin-like, have been linked to alterations in puberty.

**Objectives:**

We examined the association of peripubertal serum levels of these compounds [and their toxic equivalents (TEQs)] with pubertal onset and maturity among Russian boys enrolled at ages 8–9 years and followed prospectively through ages 17–18 years.

**Methods:**

At enrollment, 473 boys had serum dioxin-like compounds and PCBs measured. At the baseline visit and annually until age 17–18 years, a physician performed pubertal staging [genitalia (G), pubarche (P), and testicular volume (TV)]. Three hundred fifteen subjects completed the follow-up visit at 17–18 years of age. Pubertal onset was defined as TV > 3 mL, G2, or P2. Sexual maturity was defined as TV ≥ 20 mL, G5, or P5. Multivariable interval-censored models were used to evaluate associations of lipid-standardized concentrations with pubertal timing.

**Results:**

Medians (interquartile ranges) of the sum of dioxin-like compounds, TEQs, and non-dioxin-like PCBs were 362 pg/g lipid (279–495), 21.1 pg TEQ/g lipid (14.4–33.2), and 250 ng/g lipid (164–395), respectively. In adjusted models, the highest compared to lowest TEQ quartile was associated with later pubertal onset [TV = 11.6 months (95% CI: 3.8, 19.4); G2 = 10.1 months (95% CI: 1.4, 18.8)] and sexual maturity [TV = 11.6 months (95% CI: 5.7, 17.6); G5 = 9.7 months (95% CI: 3.1, 16.2)]. However, the highest compared to the lowest quartile of non-dioxin-like PCBs, when co-adjusted by TEQs, was associated with earlier pubertal onset [TV = –8.3 months (95% CI:–16.2, –0.3)] and sexual maturity [TV = –6.3 months (95% CI:–12.2, –0.3); G5 = –7.2 months (95% CI:–13.8, –0.6)]; the non-dioxin-like PCB associations were only significant when adjusted for TEQs. TEQs and PCBs were not significantly associated with pubic hair development.

**Conclusions:**

Our results suggest that TEQs may delay, while non-dioxin-like PCBs advance, the timing of male puberty.

**Citation:**

Burns JS, Lee MM, Williams PL, Korrick SA, Sergeyev O, Lam T, Revich B, Hauser R. 2016. Associations of peripubertal serum dioxin and polychlorinated biphenyl concentrations with pubertal timing among Russian boys. Environ Health Perspect 124:1801–1807; http://dx.doi.org/10.1289/EHP154

## Introduction

Considerable evidence supports a decline in age of pubertal onset among girls in recent decades (Aksglaede et al. 2009; Herman-Giddens et al. 1997; Sørensen et al. 2012), whereas studies in boys are limited with inconsistent findings (Euling et al. 2008; Herman-Giddens et al. 2012; Sørensen et al. 2012). The timing of puberty and associated physiological processes depends on neuroendocrine activation of the hypothalamic–pituitary–gonadal (HPG) axis (Havelock et al. 2004; Kronenberg et al. 2008). Developmental exposures to endocrine-disrupting chemicals (Zoeller et al. 2012) are speculated to accelerate pubertal onset in girls (Parent et al. 2015; Zawatski and Lee 2013), although a recent study reported that exposures to some persistent organic pollutants were associated with later rather than earlier pubertal onset in 645 U.S. girls (Windham et al. 2015).

Organochlorines, such as dioxin-like compounds [DLCs: polychlorinated dibenzo-*p*-dioxins, polychlorinated dibenzofurans, and dioxin-like polychlorinated biphenyls (PCBs)] and non-dioxin-like PCBs are examples of endocrine disruptors that alter pubertal timing in animal studies (Parent et al. 2015). Organochlorines are wide-spread environmental contaminants that are lipophilic and highly stable, with half-lives of many years (Milbrath et al. 2009). Consequently, even though the production of compounds such as PCBs is banned, they persist in the environment, while dioxins are still generated as by-products of industrial chemical processes and incineration (UNEP 2009). Dioxin potency is assessed by relative affinity for the aryl hydrocarbon receptor (AhR), expressed as toxic equivalents (TEQ) (Van den Berg et al. 2006).

In our previous publication on our longitudinal cohort of Russian boys, we reported follow-up to age 12 years (when 83% had pubertal onset), at which time there were associations of higher baseline serum TEQs, measured when the boys were 8–9 years, with later pubertal onset by testicular volume (TV), and a suggestion of earlier onset by TV with higher non-dioxin-like PCBs (Korrick et al. 2011). We also reported that higher maternal serum non-dioxin-like PCBs, measured when the boys were age 8–9 years, were associated with earlier pubertal onset among these boys (Humblet et al. 2011). In our present analysis, which extends follow-up to age 17–18 years, all active participants have entered puberty and the majority (95%) have completed puberty, enabling us to evaluate the associations of DLCs, TEQs, and non-dioxin-like PCBs with both pubertal onset and sexual maturity.

## Methods

### Study Population

The Russian Children’s Study is a prospective cohort study of 499 boys residing in Chapaevsk, Russia, enrolled in 2003–2005 at ages 8–9 years and followed annually through 2012–2014 to ages 17–18 years for this analysis (Hauser et al. 2005). The study was approved by the Human Studies Institutional Review Boards of the Chapaevsk Medical Association, Harvard T.H. Chan School of Public Health, University of Massachusetts Medical School, and Brigham and Women’s Hospital. The parent/guardian provided informed consent, and the boys signed assent forms before participation. For this analysis, 10 boys in the original cohort were excluded due to chronic illnesses that could affect puberty. Of the remaining 489 subjects, 473 (97%) with baseline DLC and non-dioxin-like PCB measurements were included.

At study entry, each boy’s parent or guardian completed nurse-administered health and lifestyle questionnaires (Lee et al. 2003), ascertaining birth and medical history and demographic and socioeconomic status (SES) information. A validated Russian Institute of Nutrition semi-quantitative food frequency questionnaire was used to characterize each child’s diet (Martinchik et al. 1998; Rockett et al. 1997).

### Physical Examination and Pubertal Assessment

At study entry and annual follow-up visits, a standardized anthropometric examination was performed by a single trained research nurse, and pubertal staging was performed by a single physician (O.S.) without knowledge of serum organochlorine concentrations. Staging of genitalia (G) and pubic hair (P) as 1 (immature) to 5 (sexually mature) was by visual inspection (Tanner and Whitehouse 1976). TV was measured using a Prader orchidometer. Pubertal onset was defined as G2, P2, or TV > 3 mL for either testis. Sexual maturity was defined as G5, P5, or at least one testis ≥ 20 mL (Joustra et al. 2015).

### Exposure Assessment

Fasting blood samples were collected at ages 8–9 years, and serum was stored at –35°C until shipment on dry ice to the U.S. Centers for Disease Control and Prevention (Atlanta, GA) and analyzed during 2004–2008. The samples, including method blanks and quality control samples, were analyzed for 7 dioxins, 10 furans, 41 PCBs, and total lipids as described previously (Burns et al. 2009). Values below the limit of detection (LOD) were assigned a value equal to the LOD divided by the square root of 2 (Baccarelli et al. 2005). Organochlorine concentrations were expressed as lipid-standardized concentrations (wet-weight levels divided by lipid concentrations) or as the lipid-standardized sum of the toxic-equivalency factor (TEF)–weighted dioxin-like concentrations (TEQs).

### Statistical Analysis

Unadjusted and adjusted interval-censored survival analyses were used to evaluate the associations of boys’ baseline sum of DLC (ΣDLC), TEQ (ΣTEQ), and non-dioxin-like PCB (Σnon-dioxin-like PCB) congener concentrations (in quartiles) with age at pubertal onset and sexual maturity. Ages at pubertal onset and sexual maturity were assumed to follow a normal distribution. Trend tests were conducted by modeling quartiles as an ordinal variable. The interval-censored model allows for pubertal onset and sexual maturity to occur between study visits (interval-censored), before the study entry visit (left-censored), or after the last study visit (right-censored) (Christensen et al. 2010; Lindsey and Ryan 1998). Parameter estimates using maximum likelihood methods were implemented via PROC LIFEREG in SAS version 9.2 (SAS Institute Inc.).

All covariates except height, body mass index (BMI), and parental pubertal milestones were identified as *a priori* predictors of pubertal development and were considered for inclusion in the models (Table 1). A separate model was fit for each measure of pubertal onset and sexual maturity. A core model was developed by first evaluating associations of each covariate with pubertal onset and sexual maturity and retaining those with *p* < 0.20, and then including these in a full model and using backwards selection (likelihood ratio test) to exclude covariates with *p* > 0.10. To check for confounding, covariates with *p* < 0.20 were added individually to the final model and those associated with ≥ 10% change in organochlorine trend test coefficients were retained. Statistical significance was defined as *p ≤* 0.05. Missing covariate data were addressed using a complete-case analysis.

**Table 1:** Descriptive characteristics of 473 boys with serum organochlorine measurements at entry into the Russian Children’s Study during 2003–2005.

Characteristic	Value
Physical characteristics (mean ± SD)
Height *z*-score^*a*^	0.12 ± 1.00
BMI *z*-score^*a*^	–0.19 ± 1.26
Prenatal and birth history [median (25th, 75th percentiles)]^*b*^
Mother’s age at son’s birth	22 (20, 26)
Birth weight (kg)	3.4 (3.1, 3.7)
Gestational age (weeks)	40 (38, 40)
Prenatal tobacco smoke exposure [*n* (%)]	224 (49)
Prenatal alcohol consumption [*n* (%)]	59 (13)
Boys daily dietary intake [median (25th, 75th percentiles)]^*b*^
Total calories (calories)	2,674 (2,092, 3,443)
Percent calories from carbohydrates	55 (50, 59)
Percent calories from fat	34 (30, 37)
Percent calories from protein	11 (10, 12)
Boys alcohol consumption (beer, liquor) [*n* (%)]^*b*^	274 (58)
Boys daily physical exercise level [*n* (%)]^*b*^
None	129 (27)
< 2 hr/day	142 (30)
≥ 2 hr/day	201 (43)
Household characteristics [*n* (%)]^*b*^
Parental education, maximum
Secondary education or less	37 (8)
Junior college/technical training	279 (59)
University graduate	153 (33)
Household income
< 175 U.S. dollars/month	164 (35)
175–250 U.S. dollars/month	123 (26)
> 250 U.S. dollars/month	185 (39)
Biological father resides in home	310 (66)
Serum organochlorine compounds [median (25th, 75th percentiles)]^*b*^
ΣDioxin-like compounds (pg/g lipid)^*c*^	362 (279, 495)
ΣToxic equivalents (pg TEQ/g lipid)^*d*^	21.1 (14.4, 33.2)
ΣNon-dioxin-like polychlorinated biphenyls (ng/g lipid)^*e*^	250 (164, 395)
^***a***^WHO age-adjusted *z*-scores (http://www.who.int/childgrowth/en/). ^***b***^Missing information: mother’s age at son’s birth (*n* = 5), birth weight (*n* = 3), gestational age (*n* = 4), prenatal tobacco smoke (*n* = 11), prenatal alcohol consumption (*n* = 16), dietary information (*n* = 3), boys alcohol consumption (*n* = 15), physical activity (*n* = 1), parental education (*n* = 4), household income (*n* = 1), TEQs (*n* = 5), non-dioxin-like PCBs (*n* = 5). ^***c***^Dioxin-like compounds include dioxins (TCDD, 1,2,3,7,8-PeCDD, 1,2,3,4,7,8-HxCDD, 1,2,3,6,7,8-HxCDD, 1,2,3,7,8,9-HxCDD, 1,2,3,4,6,7,8-HpCDD, OCDD), furans (2,3,7,8-TCDF, 1,2,3,7,8-PeCDF, 2,3,4,7,8-PeCDF, 1,2,3,4,7,8-HxCDF, 1,2,3,6,7,8-HxCDF, 1,2,3,7,8,9-HxCDF, 2,3,4,6,7,8-HxCDF, 1,2,3,4,6,7,8-HpCDF, 1,2,3,4,7,8,9-HpCDF, OCDF), and PCBs (IUPAC congeners 77, 81, 126, 169). ^***d***^TEQs includes dioxin-like compounds (see above) and additional PCBs (IUPAC congeners 105, 118, 156, 157, 167, 189). ^***e***^Non-dioxin-like PCBs (IUPAC congeners: 18, 28, 52, 49, 44, 74, 66, 101, 99, 87, 110, 118, 105, 151, 149, 146, 153, 138/158, 128, 167, 156, 157, 178, 187, 183, 177, 172, 180, 170, 189, 201, 196/203, 195, 194, 206).	

We and others have shown that dioxin-like compounds and non-dioxin-like PCBs have divergent associations with puberty (Humblet et al. 2011; Korrick et al. 2011; reviewed by Zawatski and Lee 2013), so we adjusted our final models for both simultaneously. We also assessed the robustness of the associations in single organochlorine models. Previously, we reported an association of non-dioxin-like PCBs with BMI and height (Burns et al. 2011) in this cohort, suggesting that these measures may mediate or confound the associations with puberty; therefore, we excluded baseline BMI and height from the primary analysis. We conducted sensitivity analyses adjusting for BMI and height *z*-scores [World Health Organization (WHO) standards] (de Onis et al. 2007). Sensitivity analyses were also conducted to assess robustness of associations after further adjustment for maternal age at menarche (available for 92% of participants).

## Results

### Study Population

Table 1 summarizes perinatal history and baseline anthropometric measurements; diet, maternal, and household characteristics; serum organochlorine concentrations; and missing data, which were minimal. The boys were, on average, within the normal range for height and BMI (de Onis et al. 2007). Correlations among the organochlorine concentrations ranged from 0.74 to 0.82 (data not shown). Baseline characteristics between those boys with (*n* = 473) versus without (*n* = 26) organochlorine measurements were similar (Burns et al. 2009). Three hundred fifteen boys completed annual follow-up visits through age 17–18 years (67% retention rate). Although serum organochlorines, height *z*-scores, and most demographic characteristics did not differ significantly between boys who remained in the study and those who dropped out before the visit at 17–18 years (data not shown), boys who remained in the study were leaner at baseline (mean BMI *z*-score, –0.34 vs. –0.05), and more likely to have postsecondary school–educated parents (95% vs. 87%).

### Pubertal Onset and Sexual Maturity

At study entry (age 8 to 9 years), pubertal onset had occurred in 9% (P2), 30% (G2), and 14% (TV > 3 mL). At age 17–18 years, all 315 boys had entered puberty, and 74% (P5), 98% (G5), and 97% (TV ≥ 20 mL) were sexually mature.

### Pubertal Onset and Organochlorines

In models co-adjusted for serum ΣTEQs and Σnon-dioxin-like PCBs (Table 2), higher quartiles of ΣTEQs were associated with later pubertal onset, with a monotonic increase in TV delay with increasing quartiles and an apparent plateau for genitalia (Table 2). In contrast, the highest Σnon-dioxin-like PCB quartile compared with the lowest was associated with earlier pubertal onset by TV, with a significant trend test. In models co-adjusted for serum ΣDLCs and Σnon-dioxin-like PCBs, the highest two quartiles of ΣDLCs compared with the first quartile were also significantly associated with later pubertal onset by TV, but not genitalia. None of the organochlorines were significantly associated with age of onset of pubarche.

**Table 2:** Adjusted mean shifts in age at pubertal onset [months (95% CI)] by quartiles (Q) of serum dioxin-like compounds (DLCs), toxic equivalents (TEQs), and non-dioxin-like polychlorinated biphenyl (PCBs) concentrations among 473 Russian boys, enrolled at ages 8–9 years and followed to 17–18 years.

Serum quartile	Testicular volume > 3 mL^*a*^		Genitalia stage ≥ 2^*b*^		Pubarche stage ≥ 2^*c*^	
	Mean shift (95% CI)	*p*-Value	Mean shift (95% CI)	*p*-Value	Mean shift (95% CI)	*p*-Value
ΣTEQs, adjusted for Σnon-dioxin-like PCBs^*d*^
Q1	Reference		Reference		Reference
Q2	4.0 (–1.9, 9.8)	0.19	8.1 (1.5, 14.7)	0.02	3.7 (–2.7, 10.1)	0.26
Q3	7.5 (0.6, 14.4)	0.03	10.1 (2.3, 17.9)	0.01	4.8 (–2.8, 12.4)	0.22
Q4	11.6 (3.8, 19.4)	0.004	10.1 (1.4, 18.8)	0.02	3.3 (–5.3, 11.8)	0.46
Trend test^*e*^		0.003		0.03		0.45
ΣDLCs, adjusted for Σnon-dioxin-like PCBs^*f*^
Q1	Reference		Reference		Reference
Q2	1.3 (–4.3, 7.0)	0.64	3.8 (–2.5, 10.1)	0.24	2.5 (–3.7, 8.7)	0.43
Q3	6.8 (0.6, 13.0)	0.03	6.5 (–0.4, 13.5)	0.07	2.5 (–4.3, 9.2)	0.48
Q4	8.1 (1.1, 15.1)	0.02	0.4 (–7.5, 8.3)	0.92	–6.4 (–14.0, 1.2)	0.10
Trend test^*e*^		0.01		0.71		0.16
ΣNon-dioxin-like PCBs, adjusted for ΣTEQs^*g*^
Q1	Reference		Reference		Reference
Q2	–3.1 (–8.9, 2.8)	0.31	0.4 (–6.2, 7.0)	0.91	–0.9 (–7.3, 5.6)	0.79
Q3	–5.6 (–12.3, 1.2)	0.11	–4.4 (–11.9, 3.2)	0.26	–5.2 (–12.7, 2.2)	0.17
Q4	–8.3 (–16.2, –0.3)	0.04	–5.3 (–14.1, 3.5)	0.24	–2.3 (–11.0, 6.5)	0.61
Trend test^*e*^		0.04		0.18		0.45
Interval-censored survival models: ^***a***^Adjusted for birth weight, household income, dietary fat intake, boy’s alcohol intake, boy’s daily exercise. ^***b***^Adjusted for birth weight, biological father living in home, parental education, daily caloric intake, boy’s alcohol intake. ^***c***^Adjusted for prenatal alcohol intake, biological father living in home, daily caloric and protein intake. ^***d***^ΣTEQ quartiles: Q1 4.0–14.5; Q2 14.6–21.0; Q3 21.1–33.2; Q4 33.3–174.7 pg/g lipid. ^***e***^Trend tests performed by modeling OC quartiles as an ordinal variable. ^***f***^ΣDLC quartiles: Q1 122–280; Q2 281–366; Q3 367–486; Q4 487–2,963 pg/g lipid. ^***g***^ΣNon-dioxin-like PCB quartiles: Q1 62–166; Q2 167–249; Q3 250–396; Q4 397–4,248 ng/g lipid.						

In single organochlorine models (see Table S1), the association of ΣTEQs with later pubertal onset by TV remained significant although attenuated: 5.6 months [95% confidence interval (CI): 0.3, 10.9] for the highest vs. lowest quartile, compared with 11.6 months (95% CI: 3.8, 19.4) when adjusted for Σnon-dioxin-like PCBs. The associations between TEQs and genitalia and between ΣDLCs and TV were attenuated, although the trend test for ΣDLCs and TV was significant. However, when modeled alone, the associations between Σnon-dioxin-like PCBs and earlier pubertal onset by TV were null (e.g. approximately 0 months).

### Sexual Maturity and Serum OC Concentrations

In models co-adjusted for ΣTEQs and Σnon-dioxin-like PCBs, higher ΣTEQs were associated with a monotonic dose–response association for later sexual maturity by TV and genitalia. However, the highest quartile of Σnon-dioxin-like PCBs compared with the lowest was associated with earlier sexual maturity by TV and genitalia (Table 3). In models co-adjusted for ΣDLCs and Σnon-dioxin-like PCBs, the highest two quartiles of ΣDLCs were significantly associated with a delay in sexual maturity by TV but not genitalia. Both higher ΣTEQs and ΣDLCs were nonsignificantly associated with a nonlinear delay in pubic hair maturation, with only the association of quartile 2 of ΣDLCs significant. Associations between Σnon-dioxin-like PCBs and mature pubic hair were null.

**Table 3:** Adjusted mean shifts in age at sexual maturity [months (95% CI)] by quartiles (Q) of serum dioxin-like compounds (DLCs), toxic equivalents (TEQs), and non-dioxin-like polychlorinated biphenyl (PCBs) concentrations among 473 Russian boys, enrolled at ages 8–9 years and followed to 17–18 years.

Serum quartile	Testicular volume ≥ 20 mL^*a*^		Genitalia stage 5^*b*^		Pubarche stage 5^*c*^	
	Mean shift (95% CI)	*p*-Value	Mean shift (95% CI)	*p*-Value	Mean shift (95% CI)	*p*-Value
ΣTEQs, adjusted for Σnon-dioxin-like PCBs^*d*^
Q1	Reference		Reference		Reference
Q2	6.0 (1.6, 10.5)	0.008	4.4 (–0.5, 9.3)	0.08	4.9 (–1.1, 10.9)	0.11
Q3	8.8 (3.7, 14.0)	< 0.001	7.5 (1.9, 13.2)	0.009	4.5 (–2.6, 11.5)	0.22
Q4	11.6 (5.7, 17.6)	< 0.001	9.7 (3.1, 16.2)	0.004	5.5 (–2.6, 13.6)	0.18
Trend test^*e*^		< 0.001		0.004		0.24
ΣDLCs, adjusted for Σnon-dioxin-like PCBs^*f*^
Q1	Reference		Reference		Reference
Q2	1.4 (–3.0, 5.8)	0.53	–0.6 (–5.5, 4.3)	0.80	6.8 (0.9, 12.8)	0.03
Q3	4.9 (0.2, 9.7)	0.04	1.1 (–4.2, 6.4)	0.68	6.2 (–0.1, 12.5)	0.06
Q4	5.5 (–0.01, 11.0)	0.05	0.2 (–6.0, 6.3)	0.96	2.5 (–4.8, 9.8)	0.50
Trend test^*e*^		0.04		0.86		0.52
ΣNon-dioxin-like PCBs, adjusted for ΣTEQs^*g*^
Q1	Reference		Reference		Reference
Q2	–5.0 (–9.4, –0.6)	0.03	–2.9 (–7.8, 2.0)	0.24	3.4 (–2.6, 9.5)	0.27
Q3	–3.0 (–8.1, 2.1)	0.25	–2.1 (–7.7, 3.6)	0.47	1.5 (–5.4, 8.4)	0.67
Q4	–6.3 (–12.2, –0.3)	0.04	–7.2 (–13.8, –0.6)	0.03	1.0 (–7.3, 9.2)	0.82
Trend test^*e*^		0.06		0.07		0.92
Interval-censored survival models: ^***a***^Adjusted for birth weight, biological father living in home, parental education. ^***b***^Adjusted for mother’s age at son’s birth, household income, daily caloric intake, boy’s daily exercise. ^***c***^Adjusted for prenatal tobacco smoke, biological father living in home. ^***d***^ΣTEQ quartiles: Q1 4.0–14.5; Q2 14.6–21.0; Q3 21.1–33.2; Q4 33.3–174.7 pg/g lipid. ^***e***^Trend tests performed by modeling OC quartiles as an ordinal variable. ^***f***^ΣDLC quartiles: Q1 122–280; Q2 281–366; Q3 367–486; Q4 487–2,963 pg/g lipid. ^***g***^ΣNon-dioxin-like PCB quartiles: Q1 62–166; Q2 167–249; Q3 250–396; Q4 397–4,248 ng/g lipid.						

In single organochlorine models (see Table S2), the associations of higher ΣTEQs with later sexual maturity remained significant although attenuated, e.g. for TV 7.7 months (95% CI: 3.6, 11.8) for the highest vs. lowest quartile, compared with 11.6 months (95% CI: 5.7, 17.6) when adjusted for Σnon-dioxin-like PCBs. Also, without adjustment for Σnon-dioxin-like PCBs, the association between ΣDLCs and TV was minimally attenuated. In contrast, without adjustment for ΣTEQs, the association between Σnon-dioxin-like PCBs and earlier sexual maturity became null (see Table S2).

### Sensitivity Analyses

**Figure 1:**
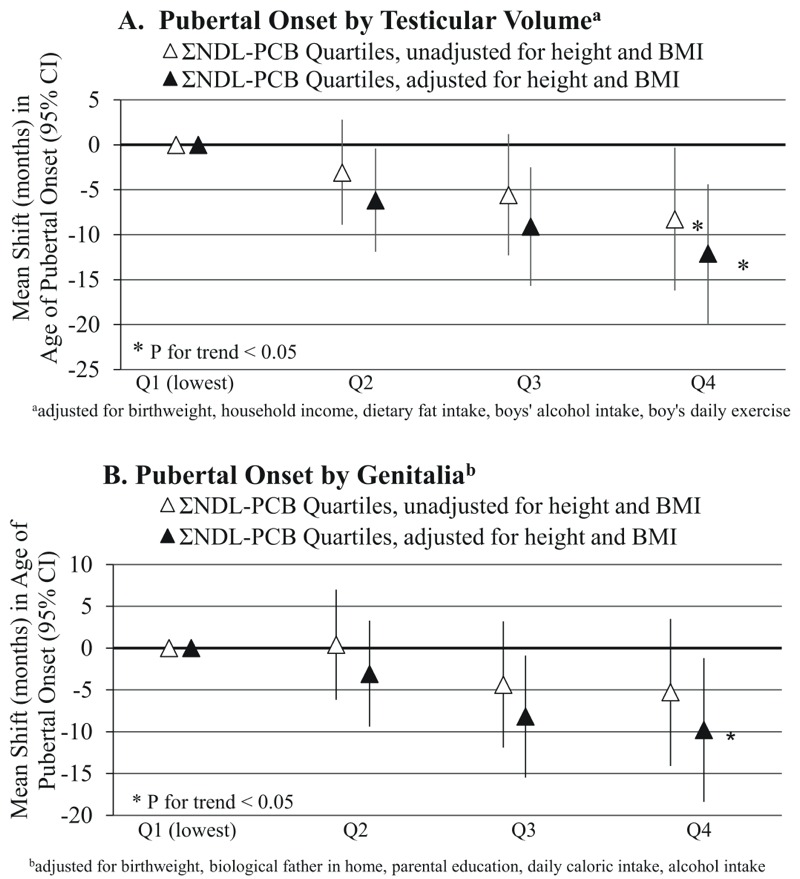
Adjusted mean shifts in age at pubertal onset [months (95% CI)] by quartiles of peripubertal serum non-dioxin-like polychlorinated biphenyl concentrations by testicular volume (*A*) and genitalia (*B*), unadjusted and adjusted for baseline body mass index and height *z*-scores.

**Figure 2:**
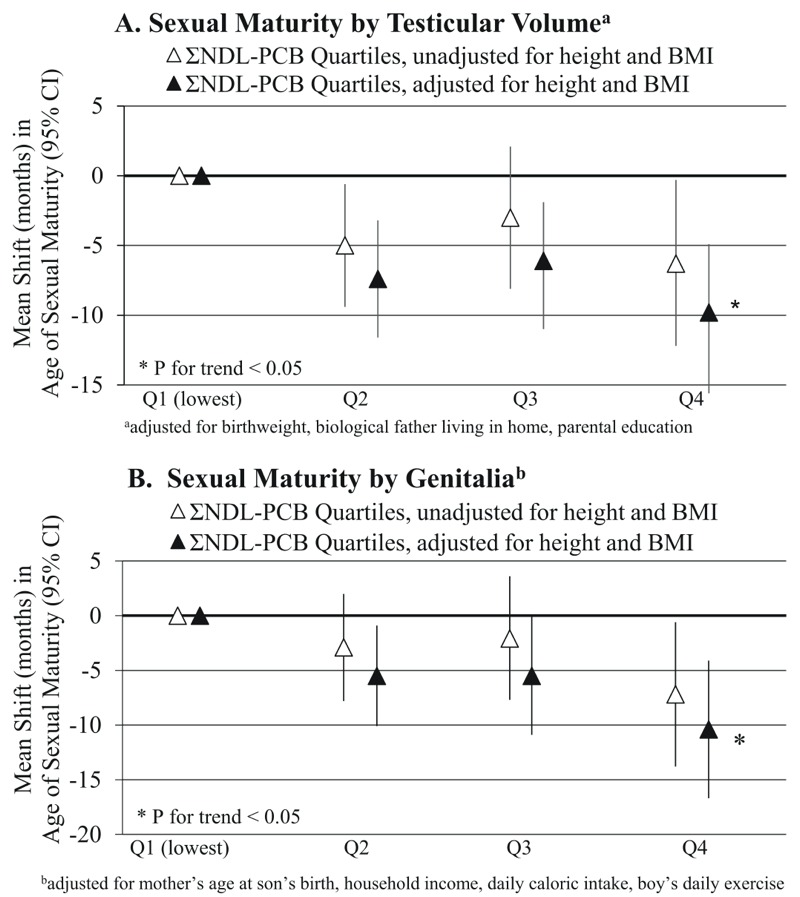
Adjusted mean shifts in age at sexual maturity [months (95% CI)] by quartiles of peripubertal serum no-ndioxin-like polychlorinated biphenyl concentrations by testicular volume (*A*) and genitalia (*B*), unadjusted and adjusted for baseline body mass index and height *z*-scores.

In multiple organochlorine models adjusted for baseline height and BMI *z*-scores, associations of ΣTEQs and ΣDLCs with pubertal onset and sexual maturity were similar to models not adjusted for height and BMI *z*-scores (see Tables S3 and S4), whereas the associations of Σnon-dioxin-like PCBs with pubertal onset and sexual maturity by TV and genitalia were strengthened (Figures 1 and 2). All associations changed minimally after adjustment for mother’s age at menarche (data not shown).

## Discussion

The few epidemiological studies that have examined the associations of dioxins and PCBs with male pubertal timing have yielded inconsistent results (Croes et al. 2014; Den Hond et al. 2002, 2011; Grandjean et al. 2012; Korrick et al. 2011; Leijs et al. 2008). In our longitudinal cohort, we examined associations among a wide range of peripubertal serum dioxin-like compounds and non-dioxin-like PCB concentrations with male pubertal onset and sexual maturity. In models with multiple organochlorines (i.e., co-adjustment), we found robust associations of dioxin-like compounds and TEQs with later pubertal onset and sexual maturity whereas non-dioxin-like PCBs were associated with earlier pubertal onset and sexual maturity.

In models co-adjusted for serum Σnon-dioxin-like PCBs, the highest compared with the lowest quartile of baseline serum ΣTEQs—the axiomatic measure of dioxin AhR toxicity—was associated with later pubertal onset and sexual maturity by TV and genitalia staging by almost a year. Similar to our results after 3 years of follow-up (Korrick et al. 2011), the present findings were robust in sensitivity analyses adjusting for baseline BMI and height *z*-scores or mother’s age at menarche (data not shown). Whereas the associations of ΣDLCs with TV were similar (albeit of a lesser magnitude) to those observed for ΣTEQs, ΣDLCs were not associated with genitalia stage. If causal, this may be consistent with a differential impact of AhR-mediated functions on TV compared with other aspects of genital maturation. Our observations of nonlinear associations of ΣDLCs with pubic hair growth may be influenced by imprecise pubic hair staging because more data were missing for this measure due to shaving pubic hair in later adolescence.

Rodent studies suggest that the potent dioxin, 2,3,7,8-tetrachlorodibenzo-*p*-dioxin (TCDD), is associated with delayed preputial separation (Bell et al. 2007; Hamm et al. 2003; Theobald et al. 2003), an androgen-dependent indicator of male pubertal onset. Few epidemiological studies have measured serum dioxins in children partly because of the large serum volume required and high cost; therefore, there are few human comparative data. A Dutch study, limited by a small sample size of 15 adolescent boys and concurrent low serum TEQs (median, 1.5 TEQ pg/g lipid), found no association between prenatal, lactational, or cross-sectional measures of serum TEQs and pubertal stage, although boys with higher cross-sectional serum TEQs reported later age at first ejaculation (Leijs et al. 2008). A Belgian group reported that living near dioxin-emitting waste incinerators as a proxy measure of exposures was associated with later pubertal maturation among 80 boys (Den Hond et al. 2002). However, both that study and later cross-sectional studies using an indirect luciferase reporter measure of AhR activity (CALUX), found no association with pubertal staging (Croes et al. 2014; Den Hond et al. 2002, 2011). Other explanations for these null findings may be attributable to indirect TEQ measurement, relatively low exposure levels, or cross-sectional design.

In contrast to our findings regarding ΣTEQs and later pubertal timing, higher peripubertal serum Σnon-dioxin-like PCBs concentrations, co-adjusted for TEQs, were associated with earlier pubertal onset by TV, and earlier sexual maturity by TV and genitalia (Tables 2 and 3). Among boys, longitudinal studies have linked clinically delayed puberty with osteopenia (Finkelstein et al. 1996) and earlier puberty with increased risk of adult obesity (van Lenthe et al. 1996) and cardiovascular disease (Prentice and Viner 2013). Alterations in timing of puberty have also been associated with psychosocial and behavior issues (Graber 2013). In both our present and prior analyses (Korrick et al. 2011), we did not observe associations between Σnon-dioxin-like PCBs and puberty in models unadjusted for ΣTEQs (see Tables S1 and S2). We ascribe this to the positive correlation between ΣTEQs and Σnon-dioxin-like PCBs (*r* = 0.82) and the strong association of ΣTEQs with later pubertal timing which may confound the effects of Σnon-dioxin-like PCBs. Thus we believe that it is necessary to include both classes of organochlorines in models assessing their associations with pubertal development. Our analytic approach was further justified by the fact that, despite correlation between measures, there was no major influence on the precision of the effect estimates for TEQs or non-dioxin-like PCBs in models including both. However, given that the biological mechanism underlying the effects of non-dioxin-like PCBs on puberty is unknown, the observed associations with puberty should be interpreted with caution.

In contrast to our assessment of peripubertal concentrations, longitudinal studies of 244 U.S. boys (Gladen et al. 2000) and 60 Taiwanese boys (Hsu et al. 2005) that assessed prenatal serum non-dioxin-like PCB concentrations did not find associations with timing of male pubertal development. However, among 394 Faroese boys, higher prenatal serum non-dioxin-like PCB concentrations were associated with a significant trend toward later pubertal development among boys at age 14 years (Grandjean et al. 2012).

Three cross-sectional Belgian studies that examined the association of peripubertal serum non-dioxin-like PCB (PCBs 138, 153, and 180) concentrations with male pubertal development by Tanner staging reported inconsistent results. Although the initial study found later pubertal development with higher serum non-dioxin-like PCBs among 80 boys (Den Hond et al. 2002), subsequently a much larger study (*n* = 887) reported a doubling of serum non-dioxin-like PCBs associated with earlier puberty (Den Hond et al. 2011). The most recent study of 324 boys 14–15 years old found no association between higher serum non-dioxin-like PCBs and pubertal timing, although higher non-dioxin-like PCBs were associated with higher serum testosterone and sex hormone-binding globulin and lower estradiol concentrations (Croes et al. 2014). In the interval between these two later studies (Croes et al. 2014; Den Hond et al. 2011), serum non-dioxin-like PCB concentrations had decreased 23%, suggesting that null findings in Croes et al. might reflect differences in exposure over time.

The inconsistent results between our study and both the earliest (Den Hond et al. 2002) and most recent (Croes et al. 2014) Belgian studies may be attributable to smaller sample size and lower serum concentrations, respectively. For example, the geometric mean (95% CI) of summed PCBs 138, 153, and 180 among the Russian boys was double that observed in Croes et al. (2014): 103.7 (95% CI: 97.4, 110.3) ng/g lipid versus 49.6 (95% CI: 45.7, 53.8) ng/g lipid. Differences in study design (cross-sectional vs. longitudinal), the unique PCB congener profile in a given community, as congeners have different estrogenic, androgenic, and anti-estrogenic properties (ATSDR 2000), and confounding by co-exposures to other endocrine disruptors are other factors that might also contribute to the inconsistent findings.

Puberty depends on a complex interplay between the central neuroendocrine system and the gonads modulated by a sex steroid–mediated negative feedback mechanism. The secretion of neuroendocrine factors stimulates the pulsatile secretion of gonadotropin-releasing hormone (GnRH), which in turn triggers pituitary secretion of luteinizing hormone (LH) and follicle-stimulating hormone (FSH) (Ebling 2005; Ojeda and Lomniczi 2014). LH stimulates testicular Leydig cells to produce testosterone, and FSH promotes the maturation of the seminiferous tubules. In concert with maturation of the HPG axis, the adrenal zona reticularis increases adrenal androgen production.

Dioxin-like compounds and PCBs can potentially perturb reproductive system homeostasis via various mechanisms, such as activation or interference with the HPG axis or inhibition of testosterone biosynthesis (Parent et al. 2015; Svechnikov et al. 2010). Experimental data show that dioxins can suppress GnRH secretion and delay pubertal onset via an AhR pathway (Mueller and Heger 2014; Parent et al. 2015). Dioxins may also have testicular actions because the AhR is widely expressed in testes (Schultz et al. 2003) and its signaling pathway has extensive crosstalk with steroid hormone receptors (Brokken and Giwercman 2014; Schultz et al. 2003).

We reported previously that higher prepubertal serum Σnon-dioxin-like PCBs concentrations were associated at ages 11–12 years with lower height and BMI (Burns et al. 2011) and that baseline height and BMI *z*-scores were associated with earlier pubertal development (Hauser et al. 2008; Korrick et al. 2011). After adjustment in our sensitivity analyses for baseline height and BMI *z*-scores, associations between Σnon-dioxin-like PCBs and earlier pubertal development were strengthened, indicating that height and BMI acted as negative confounders rather than as mediators.

We regarded our measurement of serum organochlorines at ages 8–9 years as indicative of childhood exposures, although given their long biological half-lives, these levels may also reflect perinatal exposures (Verner et al. 2015). This partially limits our ability to conclude that childhood exposures are solely responsible for the associations we observed.

Our Russian Children’s Study is one of the few prospective cohort studies to follow boys from prepuberty to sexual maturity with annual physician assessments of puberty using both Tanner staging and measurement of testicular volume, a more precise assessment than visual inspection (Biro et al. 1995). This provides the unique advantage of having more accurate data on the full pubertal process, from onset through sexual maturity. For most study participants, serum organochlorines were measured before pubertal onset, reducing the likelihood of reverse causation affecting our findings. Additionally, physical assessments were performed without knowledge of the boy’s serum organochlorine concentrations, and our analysis was adjusted for many potential confounders. Moreover, our cohort was fairly large with a high retention rate, and we found little difference between those who remained on study and those who were lost to follow-up, reducing potential bias. Our study design and analytic approach enabled us to include the observed data from all 473 boys, not just the 315 boys who completed the ninth year of follow-up, also reducing potential bias. Last, our cohort is characterized by exposure to multiple, correlated organochlorines. Given our comprehensive organochlorine measurements and our prior results (Humblet et al. 2011; Korrick et al. 2011) suggesting opposing effects, we felt the most appropriate and conservative statistical approach to assess the independent effects of TEQs and non-dioxin-like PCBs on pubertal timing was to include both in the models.

In conclusion, among boys with higher peripubertal serum levels of ΣTEQs or Σnon-dioxin-like PCBs, we observed differences in timing of puberty compared with their less-exposed peers. These findings suggest that the prepubertal window may be vulnerable to organochlorine disruption of male pubertal development. Moreover, the divergent associations we observed between ΣTEQs and Σnon-dioxin-like PCBs with later and earlier pubertal timing, respectively, may suggest that effects are mediated via different biologic mechanisms. The potential impact of these compounds on pubertal timing has implications for both adolescent and adult health, and is a critical public health concern.

## Supplemental Material

(154 KB) PDF
