# Supplementary material for: Associations of Peripubertal Serum Dioxin and Polychlorinated Biphenyl Concentrations with Pubertal Timing among Russian Boys
Source: Environ Health Perspect. 2016 May 17;124(11):1801–7. doi: 10.1289/EHP154 (PMC5089876; doi:10.1289/EHP154)
Supplement: (154 KB) PDF [file EHP154.s001.acco.pdf]

Environ Health Perspect
DOI: 10.1289/EHP154

Note to readers with disabilities: *EHP* strives to ensure that all journal content is accessible to all readers. However, some figures and Supplemental Material published in *EHP* articles may not conform to 508 standards due to the complexity of the information being presented. If you need assistance accessing journal content, please contact ehp508@niehs.nih.gov. Our staff will work with you to assess and meet your accessibility needs within 3 working days.

Supplemental Material

Associations of Peripubertal Serum Dioxin and Polychlorinated Biphenyl Concentrations with Pubertal Timing among Russian Boys

Jane S. Burns, Mary M. Lee, Paige L. Williams, Susan A. Korrick, Oleg Sergeyev, Thuy Lam, Boris Revich, and Russ Hauser

Table of Contents

Table S1. Adjusted mean shifts in age at pubertal onset [months (95% CI)] by quartiles of serum dioxin-like compounds (DLCs), toxic equivalents (TEQs), and nondioxin-like polychlorinated biphenyls (PCBs) in single organochlorine models among 473 Russian boys, enrolled at ages 8-9 years and followed up to 17-18 years

Table S2. Adjusted mean shifts in age at sexual maturity [months (95% CI)] by quartiles of serum dioxin-like compounds (DLCs), toxic equivalents (TEQs), and nondioxin-like polychlorinated biphenyls (PCBs) in single organochlorine models among 473 Russian boys, enrolled at ages 8-9 years and followed up to 17-18 years

Table S3. Adjusted mean shifts in age at pubertal onset [months (95% CI)] by quartiles of serum dioxin-like compounds (DLCs), toxic equivalents (TEQs), and nondioxin-like polychlorinated biphenyls (PCBs) among 473 Russian boys, adjusted for baseline body mass index and height z-scores, enrolled at ages 8-9 years and followed up to 17-18 years

Table S4. Adjusted mean shifts in age at sexual maturity [months (95% CI)] by quartiles of serum dioxin-like compounds (DLCs), toxic equivalents (TEQs), and nondioxin-like polychlorinated biphenyls (PCBs) among 473 Russian boys, adjusted for baseline body mass index and height z-scores, enrolled at ages 8-9 years and followed up to 17-18 years

Table S1. Adjusted mean shifts in age at pubertal onset [months (95% CI)] by quartiles of serum dioxin-like compounds (DLCs), toxic equivalents (TEQs), and nondioxin-like polychlorinated biphenyls (PCBs) in single organochlorine models among 473 Russian boys, enrolled at ages 8-9 years and followed up to 17-18 years

	Testicular Volume > 3 mL[a]		Genitalia Stage ≥ 2[b]		Pubarche Stage ≥ 2[c]	
Serum Quartile	Mean shift (95% CI)	P-value	Mean shift (95% CI)	P-value	Mean shift (95% CI)	P-value
ΣTEQs[d]						
Q1	Reference		Reference		Reference	
Q2	1.9 (-3.4, 7.3)	0.48	7.1 (1.2, 13.1)	0.02	2.3 (-3.5, 8.0)	0.44
Q3	3.2 (-2.2, 8.6)	0.24	6.9 (1.0, 12.9)	0.02	2.1 (-3.8, 7.9)	0.49
Q4	5.6 (0.3, 10.9)	0.04	5.7 (-0.2, 11.7)	0.06	0.9 (-4.8, 6.7)	0.75
Trend test[g]		0.04		0.08		0.78
ΣDLCs[e]						
Q1	Reference		Reference		Reference	
Q2	0.2 (-5.1, 5.4)	0.96	4.4 (-1.4, 10.3)	0.14	2.5 (-3.2, 8.2)	0.39
Q3	4.5 (-0.9, 9.8)	0.10	7.0 (1.0, 13.0)	0.02	3.1 (-2.7, 8.8)	0.29
Q4	4.7 (-0.5, 10.0)	0.08	1.0 (-4.8, 6.9)	0.74	-3.6 (-9.2, 2.1)	0.22
Trend test[g]		0.03		0.57		0.27
ΣNondioxin-like-PCBs[f]						
Q1	Reference		Reference		Reference	
Q2	-0.1 (-5.5, 5.2)	0.96	4.5 (-1.5, 10.4)	0.14	1.0 (-4.8, 6.8)	0.74
Q3	0.1 (-5.2, 5.4)	0.97	2.1 (-3.8, 8.0)	0.50	-2.5 (-8.3, 3.4)	0.41
Q4	0.8 (-4.6, 6.2)	0.76	2.1 (-3.9, 8.1)	0.49	0.3 (-5.6, 6.2)	0.93
Trend test[g]		0.76		0.65		0.78

Interval-censored survival models: [a]adjusted for birthweight, household income, dietary fat intake, boy's alcohol intake, boy's daily exercise; [b]adjusted for birthweight, biological father living in home, parental education, daily caloric intake, boy's alcohol intake; [c]adjusted for prenatal alcohol intake, biological father living in home, daily caloric and protein intake.
[d]ΣTEQ quartiles: Q1 4.0 – 14.5; Q2 14.6 – 21.0; Q3 21.1 – 33.2; Q4 33.3 – 174.7 pg/g lipid;
[e]ΣDLC quartiles: Q1 122 – 280; Q2 281 – 366; Q3 367 – 486; Q4 487 – 2963 pg/g lipid;
[f]ΣNondioxin-like-PCBs quartiles: Q1 62 – 166; Q2 167 – 249; Q3 250 – 396; Q4 397 – 4248 ng/g lipid.
[g]trend tests performed by modeling OC quartiles as an ordinal variable.

Table S2. Adjusted mean shifts in age at sexual maturity [months (95% CI)] by quartiles of serum dioxin-like compounds (DLCs), toxic equivalents (TEQs), and nondioxin-like polychlorinated biphenyls (PCBs) in single organochlorine models among 473 Russian boys, enrolled at ages 8-9 years and followed up to 17-18 years

	Testicular Volume ≥ 20 mL[a]		Genitalia Stage 5[b]		Pubarche Stage 5[c]	
Serum Quartile	Mean shift (95% CI)	P-value	Mean shift (95% CI)	P-value	Mean shift (95% CI)	P-value
ΣTEQs[d]						
Q1	Reference		Reference		Reference	
Q2	4.2 (0.1, 8.2)	0.04	3.1 (-1.3, 7.6)	0.17	4.9 (-0.3, 10.0)	0.06
Q3	6.1 (2.0, 10.2)	0.003	5.0 (0.5, 9.6)	0.03	3.8 (-1.4, 8.9)	0.16
Q4	7.7 (3.6, 11.8)	<0.001	4.9 (0.4, 9.5)	0.04	4.7 (-0.7, 9.6)	0.09
Trend test[g]		<0.001		0.02		0.14
ΣDLCs[e]						
Q1	Reference		Reference		Reference	
Q2	0.6 (-3.5, 4.7)	0.77	-0.4 (-4.8, 4.1)	0.88	8.0 (2.6, 13.4)	0.004
Q3	4.1 (-0.03, 8.1)	0.05	1.4 (-3.1, 6.0)	0.54	7.7 (2.4, 13.1)	0.005
Q4	5.0 (0.9, 9.1)	0.02	0.5 (-4.1, 5.0)	0.85	4.4 (-0.9, 9.6)	0.10
Trend test[g]		0.005		0.67		0.13
ΣNondioxin-like-PCBs[f]						
Q1	Reference		Reference		Reference	
Q2	-1.7 (-5.8, 2.4)	0.42	-0.2 (-4.7, 4.2)	0.92	5.7 (0.3, 11.1)	0.04
Q3	3.0 (-1.2, 7.1)	0.16	3.0 (-1.6, 7.5)	0.21	4.6 (-0.8, 10.1)	0.09
Q4	2.3 (-1.8, 6.4)	0.27	0.05 (-4.5, 4.6)	0.98	4.8 (-0.6, 10.2)	0.08
Trend test[g]		0.11		0.66		0.12

Interval-censored survival models: [a]adjusted for birthweight, biological father living in home, parental education; [b]adjusted for mother's age at son's birth, household income, daily caloric intake, boy's daily exercise; [c]adjusted for prenatal tobacco smoke, biological father living in home.
[d]ΣTEQ quartiles: Q1 4.0 – 14.5; Q2 14.6 – 21.0; Q3 21.1 – 33.2; Q4 33.3 – 174.7 pg/g lipid;
[e]ΣDLC quartiles: Q1 122 – 280; Q2 281 – 366; Q3 367 – 486; Q4 487 – 2963 pg/g lipid;
[f]ΣNondioxin-like-PCBs quartiles: Q1 62 – 166; Q2 167 – 249; Q3 250 – 396; Q4 397 – 4248 ng/g lipid.
[g]trend tests performed by modeling OC quartiles as an ordinal variable.

Table S3. Adjusted mean shifts in age at pubertal onset [months (95% CI)] by quartiles of serum dioxin-like compounds (DLCs), toxic equivalents (TEQs), and nondioxin-like polychlorinated biphenyls (PCBs) among 473 Russian boys, adjusted for baseline body mass index and height z-scores, enrolled at ages 8-9 years and followed up to 17-18 years

	Testicular Volume > 3 mL[a]		Genitalia Stage ≥ 2[b]		Pubarche Stage ≥ 2[c]	
Serum Quartile	Mean shift (95% CI)	P-value	Mean shift (95% CI)	P-value	Mean shift (95% CI)	P-value
ΣTEQs, adjusted for Σnondioxin-like-PCBs[d]						
Q1	Reference		Reference		Reference	
Q2	3.0 (-2.7, 8.6)	0.30	6.8 (0.5, 13.1)	0.04	2.7 (-3.6, 9.1)	0.40
Q3	6.1 (-0.5, 14.4)	0.07	8.3 (0.9, 15.7)	0.03	3.5 (-4.0, 11.0)	0.36
Q4	11.4 (3.9, 18.8)	0.003	9.7 (1.5, 18.0)	0.02	2.9 (-5.5, 11.3)	0.50
Trend test[g]		0.002		0.03		0.48
ΣDLCs, adjusted for Σnondioxin-like-PCBs[e]						
Q1	Reference		Reference		Reference	
Q2	-0.1 (-5.5, 5.3)	0.97	2.0 (-4.1, 8.0)	0.53	1.5 (-4.6, 7.6)	0.63
Q3	5.2 (-0.8, 11.2)	0.09	4.7 (-1.9, 11.3)	0.17	1.8 (-4.8, 8.4)	0.60
Q4	8.8 (2.1, 15.5)	0.01	0.8 (-6.8, 8.3)	0.84	-5.9 (-13.3, 1.6)	0.12
Trend test[g]		0.004		0.63		0.20
ΣNondioxin-like-PCBs, adjusted for ΣTEQs[f]						
Q1	Reference		Reference		Reference	
Q2	-6.2 (-11.9, -0.4)	0.04	-3.1 (-9.4, 3.3)	0.34	-3.1 (-9.5, 3.4)	0.35
Q3	-9.1 (-15.7, -2.5)	0.007	-8.2 (-15.5, -0.9)	0.03	-8.0 (-15.5, -0.5)	0.04
Q4	-12.1 (-19.9, -4.4)	0.002	-9.8 (-18.4, -1.2)	0.03	-5.2 (-14.0, 3.5)	0.24
Trend test[g]		0.002		0.01		0.15

Interval-censored survival models: [a]adjusted for birthweight, household income, dietary fat intake, boy's alcohol intake, boy's daily exercise; [b]adjusted for birthweight, biological father living in home, parental education, daily caloric intake, boy's alcohol intake; [c]adjusted for prenatal alcohol intake, biological father living in home, daily caloric and protein intake.
[d]ΣTEQ quartiles: Q1 4.0 – 14.5; Q2 14.6 – 21.0; Q3 21.1 – 33.2; Q4 33.3 – 174.7 pg/g lipid;
[e]ΣDLC quartiles: Q1 122 – 280; Q2 281 – 366; Q3 367 – 486; Q4 487 – 2963 pg/g lipid;
[f]ΣNondioxin-like-PCBs quartiles: Q1 62 – 166; Q2 167 – 249; Q3 250 – 396; Q4 397 – 4248 ng/g lipid.
[g]trend tests performed by modeling OC quartiles as an ordinal variable.

Table S4. Adjusted mean shifts in age at sexual maturity [months (95% CI)] by quartiles of serum dioxin-like compounds (DLCs), toxic equivalents (TEQs), and nondioxin-like polychlorinated biphenyls (PCBs) among 473 Russian boys, adjusted for baseline body mass index and height z-scores, enrolled at ages 8-9 years and followed up to 17-18 years

	Testicular Volume ≥ 20 mL[a]		Genitalia Stage 5[b]		Pubarche Stage 5[c]	
Serum Quartiles	Mean shift (95% CI)	P-value	Mean shift (95% CI)	P-value	Mean shift (95% CI)	P-value
ΣTEQs, adjusted for Σnondioxin-like-PCBs[d]						
Q1	Reference		Reference		Reference	
Q2	5.2 (1.0, 9.4)	0.02	3.1 (-1.5, 7.8)	0.18	2.9 (-2.7, 8.4)	0.32
Q3	7.5 (2.6, 14.4)	0.003	5.7 (0.3, 11.0)	0.04	1.3 (-5.2, 7.8)	0.69
Q4	11.4 (5.8, 17.0)	<0.001	8.7 (2.5, 14.9)	0.006	3.7 (-3.8, 11.2)	0.34
Trend test[g]		<0.001		0.006		0.43
ΣDLCs, adjusted for Σnondioxin-like-PCBs[e]						
Q1	Reference		Reference		Reference	
Q2	0.5 (-3.6, 4.7)	0.80	-2.0 (-6.6, 2.6)	0.39	4.7 (-0.9, 10.2)	0.10
Q3	3.7 (-0.9, 8.1)	0.11	-0.2 (-5.1, 4.8)	0.95	4.2 (-1.7, 10.1)	0.16
Q4	6.1 (0.9, 11.3)	0.02	0.5 (-5.2, 6.3)	0.96	2.3 (-4.4, 9.1)	0.50
Trend test[g]		0.02		0.75		0.51
ΣNondioxin-like-PCBs, adjusted for ΣTEQs[f]						
Q1	Reference		Reference		Reference	
Q2	-7.4 (-11.6, -3.2)	<0.001	-5.5 (-10.1, -0.9)	0.02	-0.4 (-6.1, 5.3)	0.89
Q 3	-6.1 (-11.0, -1.2)	0.02	-5.5 (-10.9, -0.04)	0.05	-2.8 (-9.3, 3.7)	0.40
Q4	-9.8 (-15.6, -4.1)	<0.001	-10.4 (-16.7, -4.1)	0.001	-3.5 (-11.2, 4.2)	0.37
Trend test[g]		0.002		0.003		0.29

Interval-censored survival models: [a]adjusted for birthweight, biological father living in home, parental education; [b]adjusted for mother's age at son's birth, household income, daily caloric intake, boy's daily exercise; [c]adjusted for prenatal tobacco smoke, biological father living in home.
[d]ΣTEQ quartiles: Q1 4.0 – 14.5; Q2 14.6 – 21.0; Q3 21.1 – 33.2; Q4 33.3 – 174.7 pg/g lipid;
[e]ΣDLC quartiles: Q1 122 – 280; Q2 281 – 366; Q3 367 – 486; Q4 487 – 2963 pg/g lipid;
[f]ΣNondioxin-like-PCBs quartiles: Q1 62 – 166; Q2 167 – 249; Q3 250 – 396; Q4 397 – 4248.
[g]trend tests performed by modeling OC quartiles as an ordinal variable.
